# Neutrophil extracellular traps contribute to myofibroblast differentiation and scar hyperplasia through the Toll-like receptor 9/nuclear factor Kappa-B/interleukin-6 pathway

**DOI:** 10.1093/burnst/tkac044

**Published:** 2022-11-16

**Authors:** Yiming Shao, Zaiwen Guo, Yunxi Yang, Lu Liu, Jiamin Huang, Yi Chen, Linbin Li, Bingwei Sun

**Affiliations:** Department of Burns and Plastic Surgery, Affiliated Suzhou Hospital of Nanjing Medical University, Suzhou 215002, Jiangsu Province, China; Department of Burns and Plastic Surgery, Affiliated Suzhou Hospital of Nanjing Medical University, Suzhou 215002, Jiangsu Province, China; Department of Burns and Plastic Surgery, Affiliated Suzhou Hospital of Nanjing Medical University, Suzhou 215002, Jiangsu Province, China; Department of Burns and Plastic Surgery, Affiliated Suzhou Hospital of Nanjing Medical University, Suzhou 215002, Jiangsu Province, China; Department of Burns and Plastic Surgery, Affiliated Suzhou Hospital of Nanjing Medical University, Suzhou 215002, Jiangsu Province, China; Department of Burns and Plastic Surgery, Affiliated Suzhou Hospital of Nanjing Medical University, Suzhou 215002, Jiangsu Province, China; Department of Burns and Plastic Surgery, Affiliated Suzhou Hospital of Nanjing Medical University, Suzhou 215002, Jiangsu Province, China; Department of Burns and Plastic Surgery, Affiliated Suzhou Hospital of Nanjing Medical University, Suzhou 215002, Jiangsu Province, China

**Keywords:** Neutrophil extracellular traps, Hypertrophic scar, Toll-like receptor 9, Fibroblast, Inflammation, Differentiation, Nuclear factor Kappa-B, Interleukin-6

## Abstract

**Background:**

Inflammation is an important factor in pathological scarring. The role of neutrophils, one of the most important inflammatory cells, in scar hyperplasia remains unclear. The purpose of this article is to study the correlation between neutrophil extracellular traps (NETs) and scar hyperplasia and identify a new target for inhibiting scar hyperplasia.

**Methods:**

Neutrophils were isolated from human peripheral blood by magnetic-bead sorting. NETs in plasma and scars were detected by enzyme-linked immunosorbent assays (ELISAs), immunofluorescence and flow cytometry. Immunohistochemistry was used to assess neutrophil (CD66B) infiltration in hypertrophic scars. To observe the entry of NETs into fibroblasts we used immunofluorescence and flow cytometry.

**Results:**

We found that peripheral blood neutrophils in patients with hypertrophic scars were more likely to form NETs (*p* < 0.05). Hypertrophic scars showed greater infiltration with neutrophils and NETs (*p* < 0.05). NETs activate fibroblasts *in vitro* to promote their differentiation and migration. Inhibition of NETs with cytochalasin in wounds reduced the hyperplasia of scars in mice. We induced neutrophils to generate NETs with different stimuli *in vitro* and detected the proteins carried by NETs. We did not find an increase in the expression of common scarring factors [interleukin (IL)-17 and transforming growth factor-β (TGF-β), *p* > 0.05]. However, inhibiting the production of NETs or degrading DNA reduced the differentiation of fibroblasts into myofibroblasts. *In vitro*, NETs were found to be mediated by Toll-like receptor 9 (TLR-9) in fibroblasts and further phosphorylated nuclear factor Kappa-B (NF-κB). We found that IL-6, which is downstream of NF-κB, was increased in fibroblasts. Additionally, IL-6 uses autocrine and paracrine signaling to promote differentiation and secretion.

**Conclusions:**

Our experiments found that NETs activate fibroblasts through the TLR-9/NF-κB/IL-6 pathway, thereby providing a new target for regulating hypertrophic scars.

## Highlights

- The infiltration of NETs in hypertrophic scars is significantly increased.
- NETs activate fibroblasts not through DNA-carried proteins but through DNA itself.
- NET DNA promotes fibroblast-to-myofibroblast differentiation through TLR-9/NF-κB/IL-6, which promotes scar hyperplasia.

## Background

Scars from trauma and surgery are still an issue for most patients. Approximately 15% of scars are difficult to heal due to abnormal growth [1]. Normal scars tend to be stable after a certain period of proliferation, the collagen bundles tend to be parallel, and finally shrink to a stable state. Pathological scars, including keloids and hypertrophic scars that show excessive dermal fibrosis, are caused by inflammatory cell infiltration, excessive cell proliferation and collagen synthesis during the wound healing process [2]. Skin tension and wound infection are major contributors to scarring. The local inflammatory response is an important factor affecting scar hyperplasia. Timely reductions in inflammation can help reduce and stabilize scar hyperplasia. Hypertrophic scars and keloids are also defined as inflammatory pathological scars [3] and various inflammatory cells are involved in the process of scar proliferation [4]. Neutrophils are the first immune cells to reach skin lesions and secrete a variety of cytokines that participate in wound healing. Neutrophils direct the pre-existing matrix to initiate repair in damaged tissues [5,6]. However, the role of neutrophils in scarring remains unclear.

Neutrophil extracellular traps (NETs) are web-like structures composed of DNA and selected cytoplasmic proteins that are released by neutrophils to entrap and kill different pathogens [7]. However, many studies have shown that overproduction of NETs may be the cause of various diseases, including tumors [8], autoimmune diseases [9,10] and multiple organ damage [11,12]. NETs carry a variety of enzymes and cytokines, which still have biological activity outside the cell and have been confirmed to be related to substantial cell damage [13–15]. The vast majority of NET-associated proteins are histones [16], with proteins from the cytoplasm and granules account for only 16% of NET-associated proteins and one third of which are elastases [17]. However, the proteins carried by NETs induced by different stimuli are also different [18]. NETs are also involved in the process of wound healing and have dual roles. Excessive neutrophil elastase (NE) degrades structural proteins in wounds, including collagen and fibronectin, which disrupt cell connections [19]. As another major protein carried by NETs, myeloperoxidase (MPO) promotes inflammation in various inflammatory diseases, leading to tissue oxidative damage [20]. The Toll-like receptors 9 (TLR-9) receptor has been reported to mediate the interaction of dissociated DNA with other cells, and NETs promote the proliferation of wound keratinocytes through the TLR-9 receptor [21]. NETs were cocultured with human skin fibroblasts and were found to increase alpha-smooth muscle actin (α-SMA) mRNA levels and collagen production. Another study also confirmed that NETs promote the differentiation of fibroblasts into myofibroblasts, thereby promoting pulmonary fibrosis [22]. Interleukin (IL)-6 is produced by various cells, including immune cells, fibroblasts, keratinocytes and endothelial cells. IL-6 produced by fibroblasts is involved in the pathogenesis of fibrosis associated with rheumatoid arthritis, progressive scleroderma, interstitial pulmonary fibrosis and lesions with abnormal wound healing such as keloids [23–25]. In a clinical study, blood IL-6 expression was significantly increased in patients with a family history of keloids [26,27]. The IL-6 signaling pathway plays an essential role in keloid pathogenesis [28,29]. Other studies have shown that downregulation of IL-6 expression in fibroblasts can significantly reduce myocardial remodeling [30].

Although NETs can promote tissue fibrosis, the mechanism remains unclear. In our study, NETs promoted the upregulation of IL-6 in fibroblasts through TLR-9/nuclear factor Kappa-B (NF-κB) and further enhanced the differentiation of fibroblasts into myofibroblasts and the secretion of collagen through IL-6, thus forming a positive feedback effect. This process eventually leads to the formation of hypertrophic scars and keloids.

## Methods

### Ethical statement

This study was approved by The Medical Ethical Committee of Nanjing Medical University. For experiments involving human blood samples, signed informed consent was obtained from all patients and healthy volunteers. Blood samples were taken from the cubital veins of patients and healthy donors. All the experimental methods were carried out in accordance with the approved guidelines. All experimental procedures involving mice were carried out in strict accordance with the recommendations in the Guide for the Care and Use of Laboratory Animals of the National Institutes of Health and State Key Laboratory of Pathogens and Biosecurity of the Institute of Microbiology and Epidemiology.

### Data collection

Blood samples from patients with scars were obtained from the Suzhou Municipal Hospital and blood samples from healthy individuals were obtained from the medical examination center. The aforementioned blood donors signed an informed consent form. For each patient, the following data were collected: age, sex, neutrophil count, neutrophil percentage, time after healing (months) and Vancouver Scar Scale (VSS) score (Table 1).

**Table 1:** Demographic and clinical characteristics of scar patients and healthy controls. The causes of injuries include burns and trauma

**Variable**	**Healthy controls**	**Patients**	***P***
Age (years)	29.9 (25–35)	33.4 (22–44)	0.0398
Gender (M:F)	13:7	15:4	0.4801
Neutrophil count (10^9^ l^−1^)	6.072 (3.4–8.4)	6.355 (4.1–10.5)	0.2806
Neutrophil percentage (%)	60.46 (50.2–68.3)	63.04 (52.7–70.4)	0.1609
Time after healing (months)	-	5.51 (3.6–10.4, SD = 1.98)	-
VSS	-	2 (1–3, SD = 0.794)	-

*M* male, *F* female, *VSS* Vancouver Scar Scale, *SD* standard deviation

### Neutrophil extraction

The neutrophil isolation kit isolates functional, highly purified neutrophils directly from human whole blood by immunomagnetic negative selection. (STEMCELL Technologies, #18103, #19666). Briefly, 50 μl of liquid A and 50 μl of liquid B (magnetic beads) were added to each 1 ml blood sample, mixed and allowed to stand for 5 min. Stem buffer diluted 1 : 1 was added to the blood sample, which was then placed in a magnetic rack and allowed to stand for 10 min. The supernatant was aspirated, 50 μl of solution B was added to each 1 ml of supernatant and the mixture was again placed in the magnetic rack for 5 min. The supernatant was aspirated and again placed in the magnetic rack for 5 min. The supernatant was then aspirated and centrifuged at 400 × g for 5 min to obtain neutrophils. Neutrophils were extracted and stored in RPMI-1640 medium (Gibco, Canada) containing 10% fetal bovine serum (FBS).

### Animals

Male C57BL/6 mice (8 weeks old, Suzhou, China) were maintained at the Animal Experimental Center of Suzhou Municipal Hospital under a 12-h light–dark cycle with free access to food and water for at least 1 week before the experiments.

The mice were anesthetized with 10% chloral hydrate by intraperitoneal injection (300 mg/kg body weight). A skin lesion with a diameter of ~1.5 cm was created on the backs of the mice. The wounds of the two groups were treated with normal saline and cytochalasin b (Cytb) every day. After the wound was healed, the healed region was collected and fixed with 4% paraformaldehyde.

### NET formation and protein purification from NETs

We inoculated 2× 10^6^ neutrophils per well in a 12-well plate with RPMI-1640 medium containing 10% fetal calf serum (FCS). The cells were allowed to settle at the bottom of the wells in the 12-well plates for 30 min before stimulation with 100 nM Phornol-12-myristate-13-acetate (PMA) for 4 h. For collection of NET, 2 ml of RPMI per well was added, and NETs (the smear on the wells) were collected in 15 ml tubes by vigorous agitation. After centrifugation at 100 × g for 5 min, NETs were collected in the supernatant and subjected to different treatments, including complete digestion by 10 U/ml DNase I (Fermentas, Germany) for 20 min at 37°C or kept undigested. The activity of DNase I was blocked with 5 mM ethylenediaminetetraacetic acid (EDTA; Applichem, GmbH, Stockholm, Sweden). Samples were sequentially centrifuged at 300 × g to remove whole cells and at 16000 × g to remove cellular debris [16].

### Quantification of MPO–DNA complexes

MPO–DNA complexes were quantified using previously described protocols [31]. The protocol used several reagents from the Cell Death Detection enzyme-linked immunosorbent assay (ELISA) kit (Roche). First, a high-binding Enzyme Immunoassay/radioimmunoassay (EIA/RIA) 96-well plate (Costar) was coated overnight at room temperature (RT) with anti-human MPO antibody (RD, DY DY3174). The plate was washed twice with wash buffer [0.05% Tween-20 in phosphate buffered saline (PBS)] and then blocked with 4% bovine serum albumin (Millipore Sigma) in PBS (with 0.05% Tween-20) for 1 h at RT. The plate was again washed five times before incubating for 90 min at RT with serum or plasma. The plate was washed five times and then incubated for 90 min at RT with anti-DNA antibody [Horseradish Peroxidase (HRP) conjugated; from the Cell Death kit]. After five more washes, the plate was developed with 3,3′,5,5′-Tetramethylbenzidine (TMB) followed by a 2 N sulfuric acid stop solution. Absorbance was measured at a wavelength of 450 nm using a Cytation 5 Cell Imaging Multi-Mode Reader (BioTek).

### Plasma histone assay

EDTA-anticoagulated blood was centrifuged at 300 × g for 10 min at RT and the top two-thirds of the plasma was carefully removed and stored at −80°C. Plasma histone levels were determined with a Total Histone H3 Sandwich ELISA Kit (#7253, Cell Signaling) according to the provided protocol. The absorbance at 450 nm was measured within 5 min to determine the histone concentration and the data were recorded.

### Western blot analysis

The extracted proteins were separated by SDS-polyacrylamide gel electrophoresis (PAGE) on 10–12% polyacrylamide gels and stained with Coomassie blue. The following primary antibodies were used: anti-NF-κB (Abcam, ab16502, 1:2000), anti-α-SMA (Abcam, ab7817; 1:2000), anti-TLR9 (Abcam, ab134268; 1:2000), anti-collagen III (Abcam, ab131260; 1:2000) and anti-IL-6 (Abcam, ab233706; 1:2000).

### Immunostaining and microscopy

Cells were fixed in 4% paraformaldehyde, permeabilized with 0.1% Triton X-100, blocked with 5% bovine serum albumin and stained with the following: anti-NF-κB (Abcam, ab16502, 1:2000), anti-α-SMA (Abcam, ab7817; 1:2000), anti-TLR9 (Abcam, ab134268; 1:2000) and anti-collagen III (Abcam, ab131260; 1:2000). 4′,6-diamidino-2-phenylindole (DAPI, Solarbio D6470) and phalloidin (Abcam, ab176759) were used to staining nucleus and cytoskeleton, respectively. The cell membrane was stained with a PKH26 Red Fluorescent Cell Linker Mini Kit (Sigma, MINI26) according to the provided protocol before the cells were fixed. After staining, the cells were observed using a Zeiss LSM 900 confocal microscope.

### Flow cytometry

After fibroblasts were cocultured with NETs, fibroblasts were stained with anti-TLR9 (Abcam, ab134268; 1:2000) and SYTOX Green (1:15000). The samples were analyzed by flow cytometry (FACSCanto II, BD Bioscience).

### Immunohistochemistry

Tissue was fixed in 4% formaldehyde, paraffin-embedded and sectioned at 5 μm. The sections were exposed to 3% hydrogen peroxide for 10 min to inhibit endogenous peroxidase activity, followed by blocking with 3% bovine serum albumin for 30 min. The tissue sections were stained with hematoxylin and eosin and then observed by light microscopy.

Tissue sections were prepared as described above and incubated with rabbit anti-mouse CD66B antibody (Abcam, ab197678; 1:2000) at 4°C overnight. The sections were then washed and incubated with anti-rabbit immunoglobulin G (IgG) secondary antibody for 30 min at RT. Then, the sections were restained with hematoxylin and developed using a diaminobenzidine kit. The results were obtained by examination under an IX73 microscope (Olympus).

Tissue sections were prepared as previously described [32]. The sections were then incubated overnight with rabbit anti-citrullinated histone 3 (citH3) antibody (Abcam, ab219407, 1:100) and rabbit anti-MPO polyclonal antibody (Abcam, ab208670, 1:100). After washing, the slides were incubated with fluorescent dye-coupled secondary antibodies for 1 h at RT. The DNA was stained with DAPI (Servicebio, G1012, 1:200) for 10 min. Images were acquired using a fluorescence microscope.

### Statistical analyses

All statistical analyses were performed and graphs were prepared with GraphPad Prism 8.0 software and Adobe Illustrator. The Shapiro–Wilk test was used to test the normality of continuous variables. The results are expressed as the mean ± standard deviation (SD). For group comparisons, one-way analysis of variance (ANOVA) was used to compare continuous variables with a normal distribution. The Kruskal–Wallis test was used to compare continuous variables with a skewed distribution. Tukey’s *post hoc* test or Dunn’s *post hoc* test was used for multiple comparisons. Student’s t test and the Wilcoxon paired signed rank test were used to compare differences between two groups. The Pearson correlation coefficient was used for correlation analysis. Significant differences are noted by asterisks (^*^p < 0.05, ^*^^*^p < 0.01, ^*^^*^^*^p < 0.001, ^*^^*^^*^^*^p < 0.0001).

## Results

### Increased NET production in the scar tissue of patients with hypertrophic scarring

**Figure 1.**
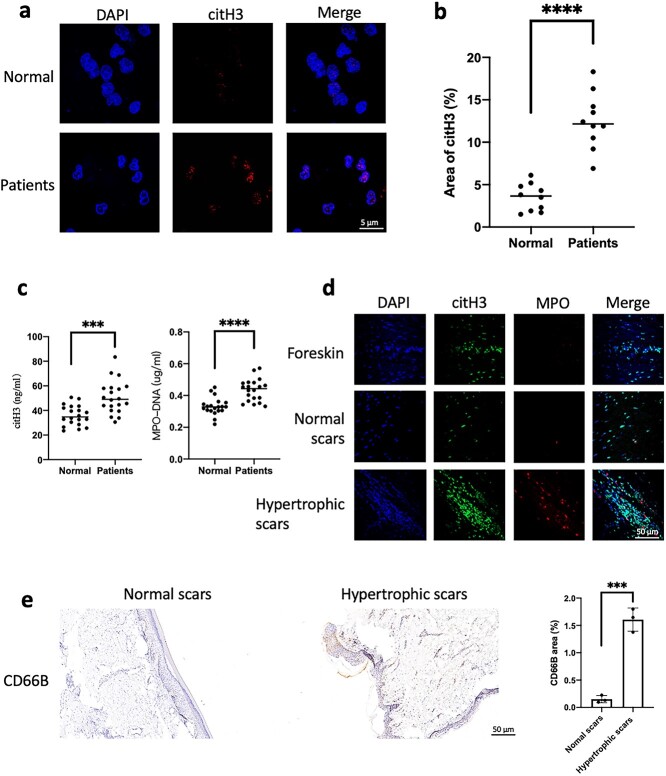
Increased production of NETs in patients with hypertrophic scarring. Peripheral blood was collected from 20 patients with scar hyperplasia and healthy volunteers (Normal group). (**a**, **b**) After purification of the neutrophils of the two groups, immunofluorescence staining of neutrophil citrullinated histones (red) was performed in the two groups (scale bar: 5 μm). The citH3 level in the neutrophil nuclei of the patient group was significantly higher than that of the healthy volunteer group (Normal group) (^*^^*^^*^^*^p < 0.0001). (**c**) ELISAs detected citH3 and NETs (MPO–DNA) in plasma, and the levels of the patient group was significantly higher than those of the healthy volunteer group (Normal group) (^*^^*^^*^p < 0.001, ^*^^*^^*^^*^p < 0.0001). (**d**) Foreskin, normal scar and hypertrophic scar tissues were sliced and stained, and the NETs (citH3 + MPO) in the hypertrophic scar increased significantly compared with those in the other two groups (scale bar: 50 μm). (**e**) Immunohistochemical staining of normal scars and hypertrophic scar tissue showed that neutrophil infiltration (CD66B) in hypertrophic scars was significantly increased compared with that in normal scars (scale bar: 50 μm, ^*^^*^^*^p < 0.001). *C* the blank control, *NETs*neutrophil extracellular traps, *citH3* citrullinated histone H3, *MPO* myeloperoxidase, *ELISAs* enzyme-linked immunosorbent assays

In this study, blood was collected from 20 healthy volunteers and patients with hypertrophic scars, and neutrophils were purified from the peripheral blood of the two groups. citH3 was increased in the nuclei of patient neutrophils (Figure 1a, b). Plasma histones and NETs (MPO–DNA) were detected. We found that circulating histones and NETs were significantly increased in the patients with hyperplasia (Figure 1c). This finding suggests that neutrophils in patients with hypertrophic scars are more likely to release NETs. We further collected foreskin, normal scar and hypertrophic scar tissues. To further assess the infiltration of NETs in the scar tissue, we sliced the above tissue and found that the infiltrating NETs in the hypertrophic scar samples increased significantly (Figure 1d). The hypertrophic scars had significantly more neutrophil infiltration than the normal scars (Figure 1e).

**Figure 2.**
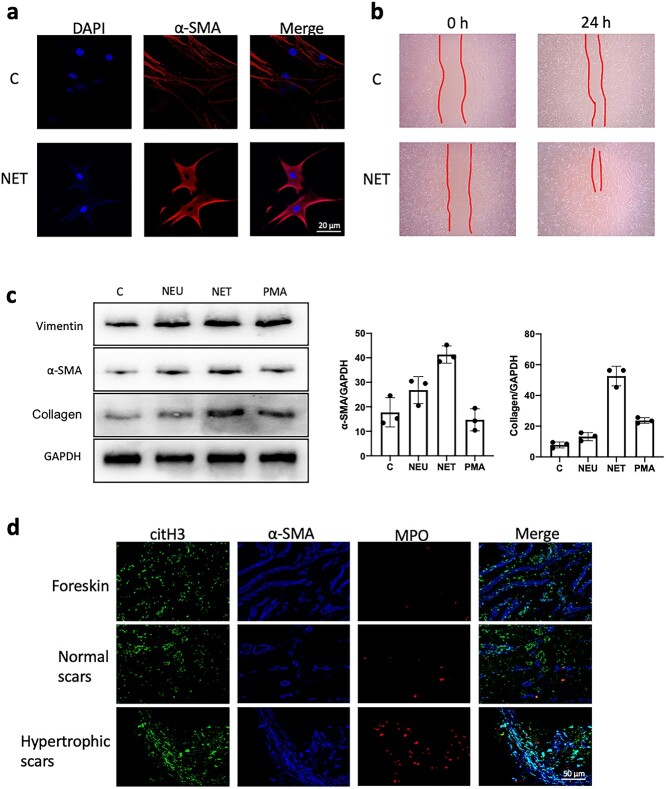
NETs stimulate fibroblast differentiation and secretion. (**a**) Fibroblasts were stimulated with NETs for 24 h *in vitro*. NETs led to a significant increase in the expression of α-SMA in fibroblasts compared with that in the control group (scale bar: 20 μm). (**b**) NET stimulation of fibroblasts for 24 h resulted in accelerated migration of fibroblasts compared to the controls. (**c**) Fibroblasts were stimulatedwith neutrophils (NEU), PMA and NET *in vitro*, and compared to no stimulation (C). WB detection showed that compared with other treatments, stimulation with NETs led to increased expression of α-SMA and collagen in fibroblasts. (GAPDH, glyceraldehyde-3-phosohate dehydrogenase) (**d**) Immunofluorescence staining of foreskin, normal scars and hyperplastic scar tissue showed increased expression and more disorganized arrangement of α-SMA (blue) within the hyperplastic scars compared with the other two groups (scale bar: 50 μm). *C* the blank control, *NETs* neutrophil extracellular traps,* α-SMA* α-smooth muscle actin,  *PMA* phorbol-12-Myristate-13-Acetate, *WB* western blot, *GAPDH* glyceraldehyde-3-phosohate dehydrogenase

**Figure 3.**
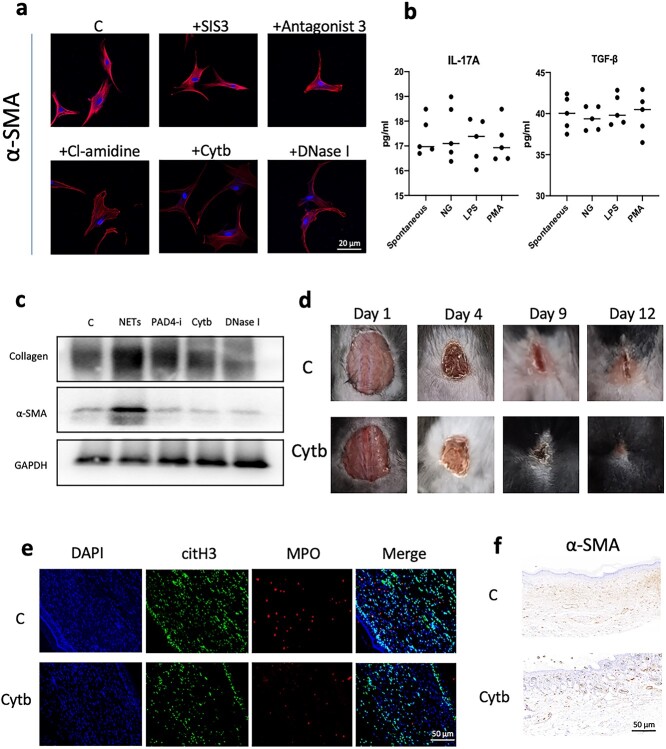
Reducing NET formation can inhibit scar hyperplasia. (**a**) Preincubation of fibroblasts with a TGF-β inhibitor (SIS3) and an IL-17A inhibitor (antagonist 3) *in vitro* did not inhibit the expression of α-SMA in fibroblasts. Inhibition or degradation of NETs with PAD4 inhibitor (Cl-amidine), cytochalasin (Cytb) or DNase I reduced the expression of a-SMA in fibroblasts (scale bar: 20 μm). (**b**) ELISA detection of NET-carrying proteins stimulated by PMA, LPS and NG on the surface of neutrophils. The amount of IL-17A and TGF-β carried on the surface was low and there was no significant difference compared with spontaneously imagined NETs. (**c**) WB analysis showed that PAD4 inhibitor (Cl-amidine), cytochalasin (Cytb) and DNase I could inhibit the expression of α-SMA and collagen in fibroblasts induced by NETs. (**d**) A skin lesion with a diameter of 1.5 cm was made on the back of the mouse and the dressings were changed with normal saline and Cytb. The wound scars of the Cytb group mice were smaller when the wound healing time was the same in the two groups of mice. (**e**) Immunofluorescence staining was performed on the back healing wound tissue of the two groups of mice and Cytb effectively inhibited the infiltration of NETs (MPO + DNA) (scale bar: 50 μm). (**f**) Immunohistochemical detection of the back healing wound tissue of the two groups of mice showed that Cytb could effectively reduce the expression of α-SMA in the tissue (scale bar: 50 μm). *C*the blank control,* NETs* neutrophil extracellular traps,*TGF-β* transforming growth factor-beta, *IL* interleukin, *α-SMA* α-smooth muscle actin, *PAD4 *peptidylarginine deiminase 4, *PMA* phorbol-12-Myristate-13-Acetate, *LPS* Lipopolysaccharide, *MPO* myeloperoxidase, *GAPDH* glyceraldehyde-3-phosohate dehydrogenase

**Figure 4.**
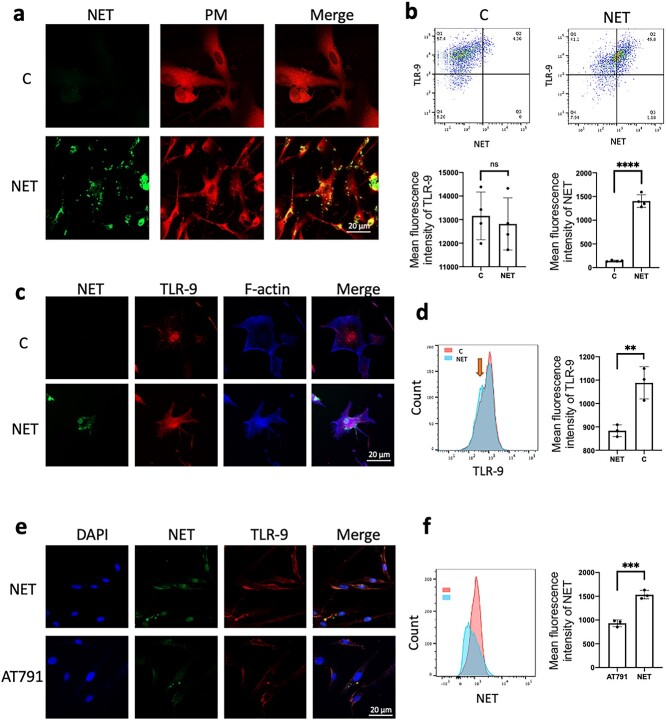
TLR-9 receptor mediates NET entry into fibroblasts. (**a**) The TLR-9 receptor mediates NET entry into fibroblasts. Fibroblasts were stimulated with stained NETs (green) for 24 h *in vitro* and some NETs entered fibroblasts (scale bar: 20 μm). (**b**) Fibroblasts were stimulated with stained NETs (SYTOX) for 24 h *in vitro* and the expression of total TLR-9 receptor and NETs in fibroblasts was detected by flow cytometry. Compared with that of the control group, the total TLR-9 receptors of fibroblasts did not change significantly (ns: *p* > 0.05) and the NETs entering the cells increased (^*^^*^^*^^*^p < 0.0001). (**c**) Fibroblasts were stimulated with stained NETs (green) for 24 h *in vitro* and the intracellular TLR-9 expression receptor was significantly increased and colocalized with the NETs that entered the cells (scale bar: 20 μm). (**d**) Flow cytometry detection of the TLR-9 receptor in fibroblast membranes. NETs stimulated for 24 h led to decreased expression of the TLR-9 receptor in fibroblast membranes (^*^^*^p < 0.01). (**e**) After preincubation of fibroblasts with a TLR-9 receptor inhibitor (AT791) *in vitro*, AT791 effectively reduced the entry of NETs into fibroblasts (scale bar: 20 μm). (**f**) NETs entered fibroblasts, as detected by flow cytometry. AT791 effectively reduced the entry of NETs into fibroblasts (^*^^*^^*^p < 0.001). *C* the blank control, *TLR-9* Toll-like receptors 9, *NETs* neutrophil extracellular traps, *ns* not statistically, *DAPI*4',6-diamidino-2-phenylindole

**Figure 5.**
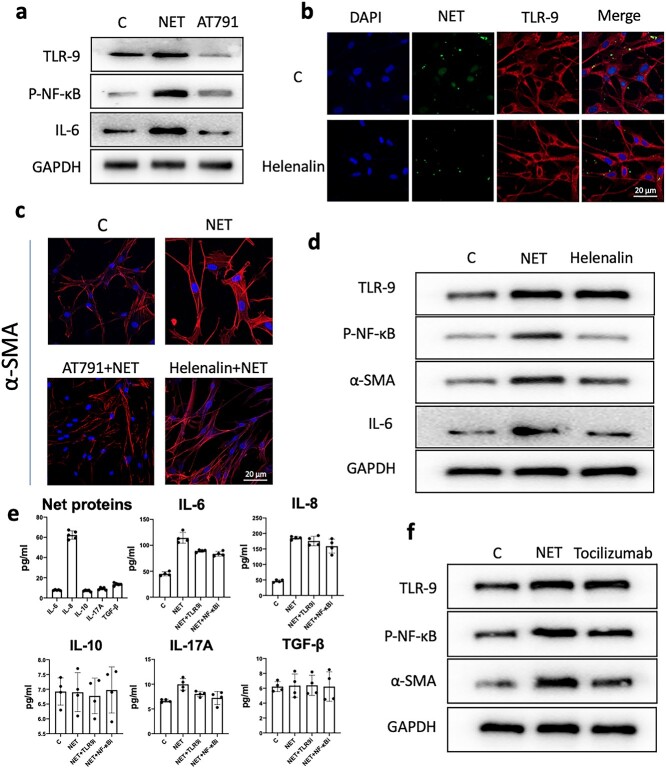
The NETs/TLR-9/NF-κB pathway mediates fibroblast IL-6 secretion and leads to scar hyperplasia. (**a**) Fibroblasts were preincubated with a TLR-9 receptor inhibitor (AT791) *in vitro* and WB detection showed that AT791 could reduce the expression of P-NF-κB and IL-6 induced by NET-stimulated fibroblasts. (**b**) Fibroblasts were preincubated with an NF-κB phosphorylation inhibitor (helenalin) *in vitro* and immunofluorescence showed that helenalin did not affect the expression of the TLR-9 receptor or the entry of NETs into fibroblasts (scale bar: 20 μm). (**c**) *In vitro* preincubation of fibroblasts with TLR-AT791 or helenalin inhibited the expression of α-SMA in fibroblasts induced by NETs (scale bar: 20 μm). (**d**) Fibroblasts were preincubated with an NF-κB phosphorylation inhibitor (helenalin) *in vitro* and WB analysis showed that helenalin did not affect the expression of the TLR-9 receptor but could reduce P-NF-κB and IL-6 in fibroblasts induced by NET-SMA expression. (**e**) PMA stimulates neutrophils to form NETs. The protein carried by NETs was purified and several cytokines were detected by ELISAs. IL-8 had the highest expression, while the expression of other cytokines was very low. After NET stimulation of fibroblasts for 24 h, the corresponding cytokines in the culture supernatant were detected and it was found that only the expression levels of IL-6 and IL-8 were increased, and IL-6 could be inhibited by AT791(TLR9i) or helenalin(NF-κBi). (**f**) Fibroblasts were preincubated with an IL-6 receptor inhibitor (tocilizumab) *in vitro*. Tocilizumab did not affect the expression of the TLR-9 receptor or P-NF-κB induced by NET stimulation but reduced the expression of α-SMA. *C *the blank control, *TLR-9 *Toll-like receptors 9, *NETs* neutrophil extracellular traps, *IL *interleukin, *α-SMA*  *α*-smooth muscle actin, *PMA* phorbol-12-Myristate-13-Acetate, *P-NF-κB* Phosphorylation nuclear factor kappa-B, *WB* western blot, *GAPDH* glyceraldehyde-3-phosohate dehydrogenase, *ELISAs* enzyme-linked immunosorbent assays, *TGF-β* transforming growth factor-*β*

**Figure 6.**
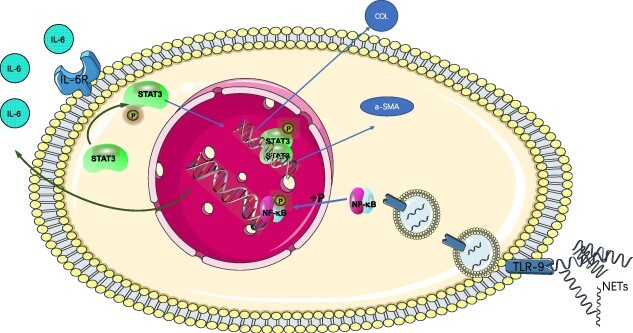
Schematic diagram of the TLR-9/NF-κB/IL-6 pathway.  NETs translocate into fibroblasts through TLR-9 receptor interactions and lead to an increase in nuclear P-NF-κB, which in turn initiates the expression and increase of IL-6 in fibroblasts. The secretion of IL-6 promotes the expression of α-SMA and the production of collagen in fibroblasts through autocrine and paracrine signaling, resulting in scar hyperplasia. *TLR-9 *Toll-like receptors 9, *NETs* neutrophil extracellular traps,* IL *interleukin, *α-SMA*  *α*-smooth muscle actin, *PMA* phorbol-12-Myristate-13-Acetate

### NETs stimulate fibroblast differentiation and secretion

When NETs were cocultured with fibroblasts *in vitro*, we found that NETs stimulated fibroblast-to-myofibroblast differentiation (Figure 2a) and promoted fibroblast migration (Figure 2b). We further examined the fibroblasts cocultured with NETs and found that the expression of α-SMA and collagen in fibroblasts was significantly increased (Figure 2c). Additionally, we stained the scar tissue and found that α-SMA was more disorganized and significantly increased in the hypertrophic scar, and the infiltration of NETs was also significantly increased (Figure 2d).

### Reducing NET formation can inhibit fibroblast-to-myofibroblast differentiation

Before coculture with NETs, we preincubated fibroblasts with inhibitors of transforming growth factor-β (TGF-β), IL-17A, PAD4 (peptidylarginine deiminase 4), cytochalasin and DNase I. We found that inhibitors of TGF-β and IL-17A did not inhibit NET-induced differentiation, but inhibition of NET production or DNA degradation could effectively inhibit fibroblast differentiation (Figure 3a). Given that TGF-β and IL-17A are the main factors that stimulate scarring, we extracted the proteins carried by NETs and found that NETs had very low levels of TGF-β and IL-17A (Figure 3b). We analyzed fibroblast proteins simultaneously. Inhibiting NETs or degrading DNA effectively inhibited fibroblast-to-myofibroblast differentiation and collagen secretion (Figure 3c). We created lesions ~1.5 cm in diameter on the backs of mice. Mice were divided into control group (C) and cytochalasin group (Cytb), and the dressings were changed with saline or Cytb (5 mg/kg), respectively. We found that moderate amounts of Cytb could effectively inhibit scar hyperplasia (Figure 3d). In previous studies, we showed that Cytb can effectively inhibit the generation of NETs. We stained the dorsal healing site of mice and found that Cytb effectively inhibited the infiltration of NETs in the scar (Figure 3e). Immunohistochemistry also showed that Cytb could effectively inhibit the differentiation of fibroblasts to myofibroblasts in the back scars of the mice (Figure 3f).

### The NET/TLR-9/NF-κB pathway mediates fibroblast IL-6 secretion and leads to scar hyperplasia

After coincubating NETs with fibroblasts for 24 h, we found that some NETs entered fibroblasts (Figure 4a). TLR-9 receptors have been shown to mediate various cellular interactions with cell-free DNA and DNA translocation into cells. We analyzed total TLR-9 receptors and NETs in fibroblasts by flow cytometry and found that NETs in fibroblasts increased, but total TLR-9 receptors did not change significantly (Figure 4b). This finding is also similar to the results of immunofluorescence analysis of cells (Figure 4c). Interestingly, we found that the stimulation of fibroblasts by NETs resulted in decreased membrane TLR-9 receptors (Figure 4d).

TLR-9 mediates the activation of NF-κB in a variety of cells and promotes the secretion of inflammatory factors. Fibroblasts were preincubated with a TLR-9 receptor inhibitor (AT791) before coincubation of NETs with fibroblasts for 24 h. We found that AT791 not only reduced the expression of TLR-9 after NET stimulation but also reduced the activation of NF-κB (Figure 5a). Furthermore, we preincubated fibroblasts with an inhibitor of NF-κB phosphorylation (helenalin) before coincubating NETs with fibroblasts. Helenalin effectively inhibited the entry of NETs into cells (Figure 5b). Immunofluorescence staining also showed that AT791 and helenalin could effectively inhibit the expression of α-SMA in fibroblasts (Figure 5c). Western blots (WBs) also showed that helenalin could effectively inhibit the differentiation of fibroblasts into myofibroblasts and the secretion of collagen (Figure 5d). To search for factors downstream of the TLR-9/NF-κB pathway, we first detected the proteins carried by NETs. We found that only IL-8 was carried more than other inflammatory factors. We tested the culture supernatant of NETs incubated with fibroblasts for 24 h and found that the secretion of IL-6 and IL-8 was significantly increased (Figure 5e). AT791 and helenalin effectively inhibited the secretion of IL-6 in fibroblasts. We further preincubated fibroblasts with an IL-6 receptor neutralizing antibody (tocilizumab) and found that the IL-6 neutralizing antibody could effectively inhibit fibroblast differentiation and protoprotein secretion (Figure 5f). The above results showed that the NET/TLR-9/NF-κB pathway mediates fibroblast IL-6 secretion and leads to scar hyperplasia (Figure 6).

## Discussion

Scar formation is the final stage of wound healing, which involves the degeneration of neovascularization and concomitant reconstruction of the extracellular matrix (ECM), resulting in the development of organized collagen fibrils that serve as the basis of normal scarring [33]. When these processes are disturbed, pathological healing results in an ulcerative chronic wound, a hypertrophic scar or a keloid [34]. Fibroblasts and myofibroblasts are the main cell types responsible for the rearrangement and secretion of the ECM and directly determine whether there is scarring in injured tissue [35,36]. Scar formation is attributed to the interaction of inflammatory cells and fibroblasts during wound healing. Neutrophils play an important role in the early stage of wound healing. This study found that the infiltration of neutrophils in hypertrophic scars was significantly higher than that in normal scars. We found that the infiltration of NETs in hypertrophic scars increased significantly, as shown by fluorescent staining of scar tissue sections. Additionally, we found that neutrophils are more likely to form NETs in patients with scar hyperplasia. Therefore, we conclude that the infiltration of NETs is closely related to scar hyperplasia.

Fibroblasts provide ECM substances, such as collagen, fibronectin, glycosaminoglycans, proteoglycans and hyaluronic acid [37,38]. The differentiation of fibroblasts into myofibroblasts is an important marker of scar maturation and plays a role in shrinking the wound surface. We found that NETs can effectively promote the differentiation of fibroblasts into myofibroblasts and reduce the expression of collagen *in vitro*. Additionally, we found that NETs can promote fibroblast migration. The above results indicate that NETs can activate fibroblasts *in vitro* and may be one of the reasons for scarring.

Activated neutrophils use multiple strategies, including phagocytosis, autophagy, degranulation, reactive oxygen species (ROS) release and NET formation, to destroy infectious threats [39]. NETs are composed of DNA carrying a variety of enzymes and cytokines, which may be involved in the activation of fibroblasts [40]. NETs are composed of histones, followed by granular enzymes and peptides, including NE, MPO, cathepsin G, leukocyte proteinase 3, lactoferrin, gelatinase, lysozyme C, calprotectin, neutrophil defensins and cathelicidins [16]. Proteases, including MPO and NE, are believed to play a major role in the bactericidal process of NETs [7,41,42]. However, NETs have been found to be associated with a variety of diseases and are closely related to the proteins carried by NETs. For example, histones are associated with vascular disease [13,43] while MPO and NE play an important role in tissue damage in a variety of diseases [44,45]. Therefore, we wondered whether NETs activate fibroblasts through the proteins they carry.

IL-17 and TGF-β are the main factors reported to activate fibroblasts. We found that the amounts of IL-17 and TGF-β carried by NETs did not increase, and inhibitors of IL-17 and TGF-β did not inhibit the activation of fibroblasts by NETs. After we used DNase I to degrade DNA, the remaining protein did not activate fibroblasts. Additionally, we found that inhibiting the generation of NETs effectively inhibited fibroblast activation, suggesting that DNA is the main mediator of fibroblast activation. In our previous study, we found that Cytb can effectively inhibit the formation of NETs and inhibit the infiltration of NETs in the lungs of septic mice [12]. Therefore, we suspect that DNA activates fibroblasts in some way. Interestingly, we found that the NETs could be transferred into fibroblasts when they were cocultured with fibroblasts.

TLR-9 has been found to mediate the entry of dissociated DNA into a variety of cells and further activate downstream signaling within the cells [46,47]. Our study found that TLR-9 receptors mediate the entry of NETs into fibroblasts, which can be inhibited by TLR-9 neutralizing inhibitors. Rapid activation of NF-κB is critical for immune responses because all TLR signaling pathways result in NF-κB activation, controlling the expression of many inflammatory cytokine genes [48,49]. In our experiments, we found that TLR-9 activates NF-κB in fibroblasts. NF-κB phosphorylation inhibitors cannot inhibit NET entry into cells but effectively inhibit the differentiation of fibroblasts into myofibroblasts and the secretion of collagen. IL-6 is a key cytokine with a proinflammatory function and its mediated inflammatory response is a key factor in the pathogenesis of keloids [28]. In a comparison of proteins carried by NETs and coculture supernatant proteins, we found that IL-6 secretion increased after NET stimulation of fibroblasts. Thus, we found that NETs activate the TLR-9 receptor on fibroblasts and translocate into the cells to further activate NF-κB, resulting in increased IL-6 secretion. IL-6 further activates fibroblasts to secrete collagen and differentiate into myofibroblasts through autocrine and paracrine signaling.

## Conclusions

Scar maturation is one of the components of wound healing. Neutrophils are involved in the complete process of wound healing. However, the current research on neutrophils mainly focuses on the early stage of wound healing. In the late stage of wound healing, especially the scar hyperplasia stage, the effect of neutrophils on the scar is not clear. Our study found that NETs mediate scar hyperplasia through the TLR-9/NF-κB/IL-6 pathway, providing a new target for the treatment of hypertrophic scars. We further elucidated the mechanism by which neutrophils affect wound scarring through NETs.

## Abbreviations

citH3: Citrullinated histone 3; Cytb: Cytochalasin b; ECM: Extracellular matrix; MPO: Myeloperoxidase; NETs: Neutrophil extracellular traps; RT: Reverse transcription; α-SMA: α-Smooth muscle actin; WB: Western blot.

## Data availability

Data are available from the authors upon reasonable request.

## Authors’ contributions

YS, ZG and BS designed the study and wrote the paper. YS, ZG, YY and LLiu performed the experiments. ZG, YC, LLi and JH collected the clinical data. YS and BS performed the statistical analysis. All authors read and approved the final manuscript.

## Ethics approval and consent

This study was approved by The Medical Ethical Committee of Nanjing Medical University. For experiments involving human blood samples, signed informed consent was obtained from all patients and healthy volunteers. Blood samples were taken from the cubital veins of patients and healthy donors. All the experimental methods were carried out in accordance with the approved guidelines. All experimental procedures involving mice were carried out in strict accordance with the recommendations in the Guide for the Care and Use of Laboratory Animals of the National Institutes of Health and State Key Laboratory of Pathogens and Biosecurity of the Institute of Microbiology and Epidemiology.

## Funding

This study was supported by the National Natural Science Foundation of China, (No. 82072217, 81772135 and U21A20370) and by the Jiangsu Natural Science Foundation (No. BK20201178).

## Conflicts of interest

The authors declare that they have no competing interests.

## Supplementary Material

Responses_to_Reviewers-R2_tkac044
