# Supplementary material for: Neutrophil extracellular traps contribute to myofibroblast differentiation and scar hyperplasia through the Toll-like receptor 9/nuclear factor Kappa-B/interleukin-6 pathway
Source: Burns Trauma. 2022 Nov 16;10:tkac044. doi: 10.1093/burnst/tkac044 (PMC9668674; doi:10.1093/burnst/tkac044)
Supplement: Responses_to_Reviewers-R2_tkac044 [file responses_to_reviewers-r2_tkac044.docx]

**Responses to Reviewer**

1．I cannot comment on Figure 3 as it is missing and figure 1 is duplicated in its place.

AU: We appreciate the reviewer’s reminding. We have reedited the text and added Figure 3 in the revised manuscript.

2．The authors in several places state they have shown that NETS induce hyperplasia, but for the in vitro work what they show are effects on fibroblast differentiation. They must be more precise and change the subheading titles to fibroblast differentiation.

**AU:** Thanks for your suggestion. The subheading titles has been changed as: " Reducing NET formation can inhibit fibroblast-to-myofibroblast differentiation" in the revised manuscript.

3. As a result of my comments in 2 above I think that the title should be changed to "Neutrophile extracellular traps contribute to myofibroblast differentiation through the TLR-9/NF-κB/IL-6 pathway".

**AU:** Thanks so much for your valuable advice. Fibroblasts are one of the most important cells in scar. Differentiation of fibroblasts into myofibroblasts and secretion of collagen is the main cause of scarring (1-3). According to your valuable suggestion, the title of the revised manuscript has been changed as “Neutrophile extracellular traps contribute to myofibroblast differentiation and scar hyperplasia through the TLR-9/NF-κB/IL-6 pathway”

**References:**

1. Qian H, Shan Y, Gong R, Lin D, Zhang M, Wang C, et al. Fibroblasts in Scar Formation: Biology and Clinical Translation. Oxid Med Cell Longev. 2022;2022:4586569.

2. Moretti L, Stalfort J, Barker TH, Abebayehu D. The interplay of fibroblasts, the extracellular matrix, and inflammation in scar formation. J Biol Chem. 2022;298:101530.

3. Jiang D, Rinkevich Y. Distinct fibroblasts in scars and regeneration. Curr Opin Genet Dev. 2021;70:7-14.

4.Figure 1D - The DAPI staining in the normal scar section cannot be seen, this must be improved. The hypertrophic scar label needs to be in the same font as the rest of the text.

AU: We thanks for your reminding. We modified the brightness of the DPAI in Figure 1d, and corrected the font of the “hypertrophic scar”.


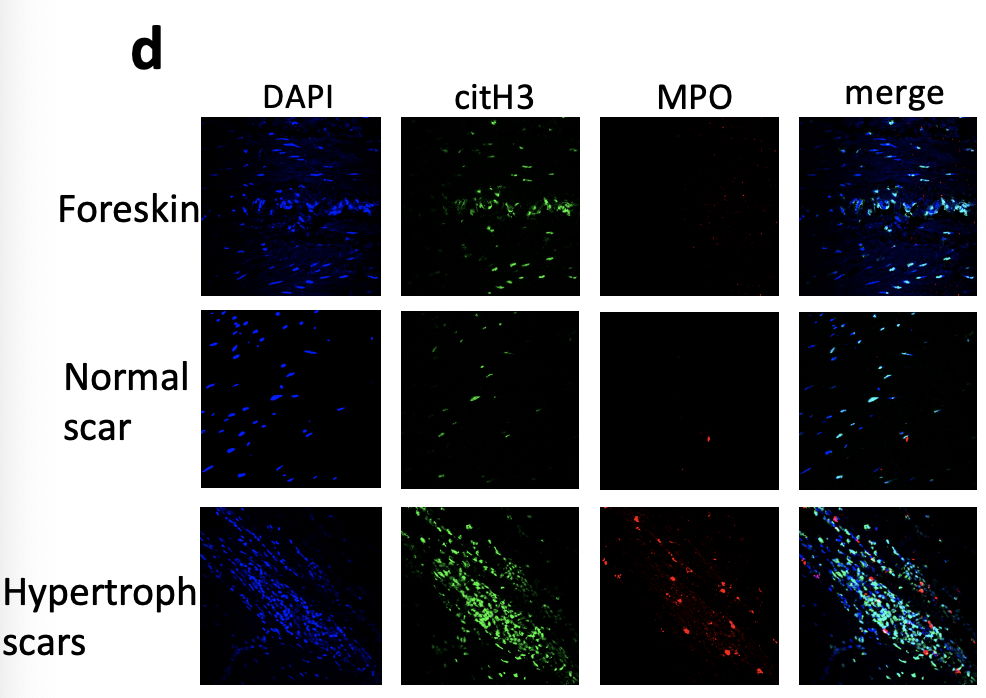


5.Figure 2A, there is still only one cell in the control image. More must be added.

AU: Thanks for your suggestion. The right pictures have been used in the revised manuscript.


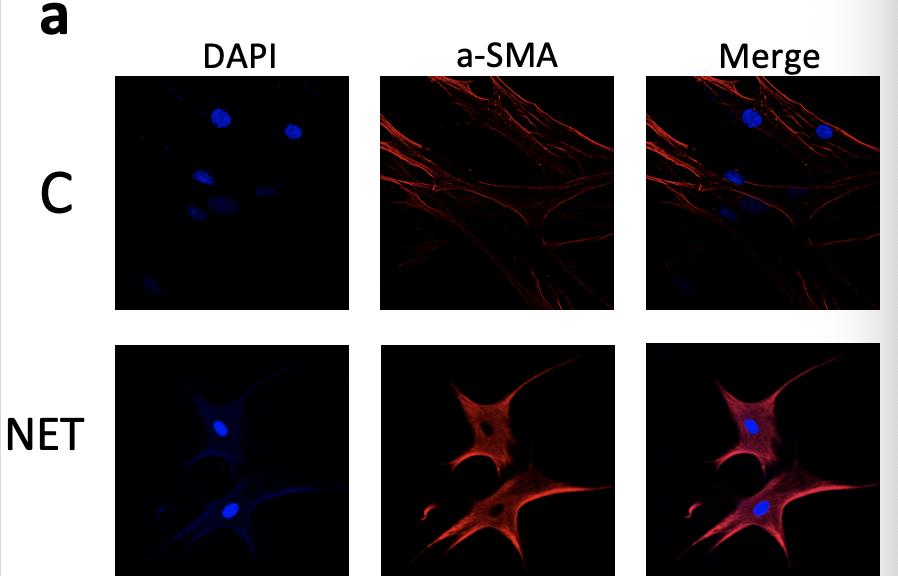


6. the abstract method should be improved to a narrative rather than a list of tests

AU: Thanks for your suggestion. Based on your suggestion, the expression of the methods in the abstract section has been revised.
